# Insight into the interactions, residue snorkeling, and membrane disordering potency of a single antimicrobial peptide into different lipid bilayers

**DOI:** 10.1371/journal.pone.0187216

**Published:** 2017-11-10

**Authors:** Majid Jafari, Faramarz Mehrnejad, Farahnoosh Doustdar

**Affiliations:** 1 Department of Life Sciences Engineering, Faculty of New Sciences & Technologies, University of Tehran, Tehran, Iran; 2 Department of Microbiology, Faculty of Medicine, Shahid Beheshti University of Medical Sciences, Tehran, Iran; Nanyang Technological University, SINGAPORE

## Abstract

Pardaxin, with a bend-helix-bend-helix structure, is a membrane-active antimicrobial peptide that its membrane activity depends on the lipid bilayer composition. Herein, all-atom molecular dynamics (MD) simulations were performed to provide further molecular insight into the interactions, structural dynamics, orientation behavior, and cationic residues snorkeling of pardaxin in the DMPC, DPPC, POPC, POPG, POPG/POPE (3:1), and POPG/POPE (1:3) lipid bilayers. The results showed that the C-terminal helix of the peptide was maintained in all six types of the model-bilayers and pardaxin was tilted into the DMPC, DPPC, and POPG/POPE mixed bilayers more than the POPC and POPG bilayers. As well as, the structure of zwitterionic membranes was more affected by the peptide than the anionic bilayers. Taken together, the study demonstrated that the cationic residues of pardaxin snorkeled toward the interface of lipid bilayers and all phenylalanine residues of the peptide played important roles in the peptide-membrane interactions. We hope that this work will provide a better understanding of the interactions of antimicrobial peptides with the membranes.

## 1. Introduction

Understanding the interactions of transmembrane proteins with lipid bilayers, their energetically properties and mechanism of actions are one of the important questions that engaged researchers for a long time ago. During the past decades, many experimental and MD simulation studies have conducted to examine and indicate the complexity of the membrane-protein interactions [1–9]. Previous studies have shown that the existence of arginine (Arg) and lysine (Lys) residues in the hydrophobic core of the bilayers is energetically expensive. However, there are transmembrane proteins with cationic residues in the hydrophobic core of plasma membrane, such as voltage-gated potassium channels and integrin proteins. This high energetic cost can decrease with hydration of the cationic side chain by snorkeling of the charged residues toward the membrane surface and penetration of water molecules as a result of the local lipid bilayer deformation [1–5]. All membrane proteins have the helical structures with a length of 4nm except for mitochondrial or bacterial outer membrane beta-barrel pores [3]. Therefore, investigation of physicochemical properties, conformational and dynamics changes of a helical peptide and behavior of its residues into membrane ambient could help us to understand the interactions of transmembrane proteins with lipid bilayers.

There are an immense number of cell penetrating, hemolytic, and antimicrobial peptides (AMPs) that have discovered during the past decades [10–14]. From the structural standpoint, many of these peptides were classified into the same class and could form an amphiphilic helical structure on the surface of bilayers [15]. Many of AMPs and cell penetrating peptides (CPPs) are enriched in the Lys and/or Arg residues [3,15], and can differ in charge, length, amino acid sequences, and mechanism of actions on the target membrane.

Although some of AMPs such as brevinin-1TP4 [16], TP4 [17], and HsAp1[18] have anti-mammalian cell activities, they are active against cancer cells, gram-positive and gram-negative bacteria, fungi, viruses, and parasites [16–25]. AMPs do not have any specific receptor on the surface of cell membrane. On the other hand, they directly affect the lipid bilayer of target cells, hence, harmful micro-organisms could not develop the AMP resistance strains [26]. Accordingly, AMPs are a suitable choice as a new generation of antibiotics.

Aggregation of AMPs on or into the phospholipid bilayers can lead to membrane perturbation or membrane disruption using different mode of actions. Alamethicin, melittin, and maginine [26–31] are pore-forming peptides, of which a certain number of the peptides aggregate in the lipid bilayers to form a barrel-stave channel or a toroidal pore, leading to disruption of ion hemostasis and cellular death in the target cells. Previous studies have shown that pardaxin forms cylindrical channels into bilayers with only single size [32]. Therefore, it would be interesting to study of pardaxin behavior into different membranes in detail.

Pardaxins are a class of antimicrobial peptides with approximately 3.5 KDa molecular weight and a bend-helix-bend-helix secondary structure motif composed of 33 amino acids [33–36]. They belong to the pore-forming AMPs family and act directly on the plasma membrane [37,38]. Pardaxins have bactericidal effects on the gram-positive and gram-negative bacteria higher than other AMPs such as dermaseptins, cecropins, and magainin [39,40]. Additionally, the peptides have anticancer activities and less hemolytic activity than melittin [36,40]. All known pardaxins were extracted from *Pardachirus pavonius* (western Pacific Peacock sole) and *Pardachirus marmoratus* (Red Sea Moses sole) and were named as P1 to P5. The amino acid sequence of this peptide family is similar to each other except the point mutations at positions 5, 14 or 31 [34]. The peptides have a hydrophobic N-terminal with an alpha helix (2–10 residues), an amphipathic C-terminal helix (13–26 residues), and a hinge with proline at position 13, which is essential for the antimicrobial activity [34,35,38,41].

In this study, P4 (sequence: GFFALIPKIISSPLFKTLLSAVGSALSSSGGQE) was selected [35]. We conducted six MD simulations for the peptide in the 1, 2-dimyristoyl-phosphatidylcholine (DMPC), 1, 2-dipalmitoylphosphatidylcholine (DPPC), 1-palmitoyl-2-oleoyl-phosphatidylcholine (POPC), and 1-palmitoyl-2-oleoyl-phosphatidylglycerol (POPG) lipid bilayers. Pardaxin is an AMP with high antibacterial activities; we also aimed to investigate the effects of the peptide on the bacterial model membranes. Therefore, we have also selected two models of the membrane including 1-palmitoyl-2-oleoyl-phosphatidylglycerol/1-palmitoyl-2-oleoyl phosphatidylethanolamine (POPG/POPE) with a ratio of 3:1 (a model for the gram-negative bacteria) and POPE/POPG with a ratio of 1:3 (a model for gram-positive bacteria) [42,43]. We investigated the structural and dynamical changes of the peptide, the pardaxin-bilayer interactions in detail, and the membrane responses to the peptide.

## 2. Materials and methods

The initial coordinates for pardaxin were obtained from protein data bank (PDB ID: 1XC0)[35]. The starting membrane coordinates of POPG were taken from Lipidbook [44] and the initial coordinates and topologies of DMPC, DPPC, and POPC bilayers were obtained from the Tieleman laboratory [45]. The POPG/POPE mixed bilayers were taken from previous studies [46,47]. All pure lipid bilayers were simulated for 100 ns and the last snapshot of each system was used as the initial point for peptide-bilayer simulations.

**Fig 1:**
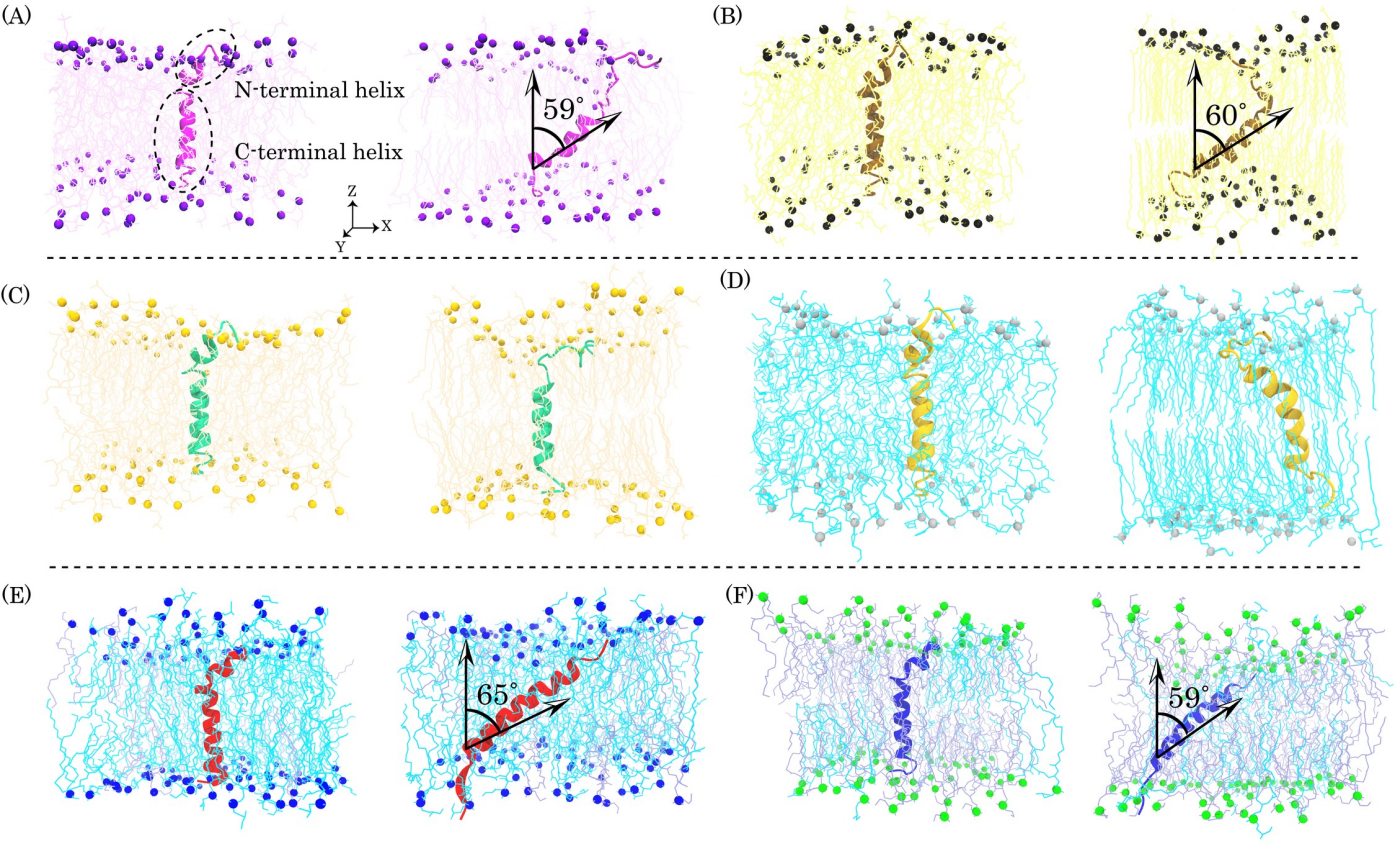
**Represents the initial (left column) and last (right column) snapshots of bilayer-peptide systems in the (A) DMPC, (B) DPPC, (C) POPC, (D) POPG, (E) POPG/POPE (3:1), and (F) POPG/POPE (1:3) systems**. In all models membrane the N-terminal and C-terminal helices of pardaxin are embedded in the top and bottom leaflets, respectively. Phosphorus atoms are shown as CPK model and water molecules and ions are not shown for clarity.

A transmembrane orientation for the peptide in the phosphatidylcholine lipid bilayers was reported in previous NMR studies [35,48]. Therefore, to mimic the peptide orientation after insertion into the lipid bilayers, the initial direction of pardaxin was set perpendicular to the surface of the model lipid membranes. The N- and C-terminal domains of the peptide were embedded in the upper and lower leaflets, respectively (Fig 1). All MD simulations were conducted using the GROMACS 4.6.5 package [49] and the GROMOS96 53A6 force field [50]. The SPC water model [51] was used and to neutralize all simulation systems, the appropriate numbers of the negatively charged CL^-^ ions and positively charged Na^+^ ions were added to each unit cell. The steepest descent algorithm was used for energy minimization and each system was then equilibrated by an NVT ensemble for 1000 ps. All systems were subsequently run using an NPT ensemble at 310 K and 1 bar of pressure. The temperature and pressure in the NPT equilibration were controlled by the Nose-Hoover thermostat [52,53] and the Parrinello-Rahman barostat [54], respectively. All covalent bonds were constrained by the LINCS algorithm [55] with a time step of 2 fs. The Particle Mesh Ewald (PME) algorithm [56] was used for calculating the long-range electrostatic interactions. A distance cutoff of 1.2 nm was applied for the short-range nonbonded interactions. Periodic boundary conditions were used for all MD simulation systems. The bilayer thickness analysis was carried out using GridMAT-MD software [57] and the van der Walls energies were calculated using the MM/PBSA method with g_mmpbsa software [58,59]. The simulation length for each peptide-bilayer system, each pure bilayer, and pardaxin in water was 500 ns, 100 ns, and 200 ns, respectively. For each of the six MD simulations, two independent trajectories have been obtained and the analyses have been done from all two independent trajectories. At the same time, the reported data contain analysis from one of the two trajectories.

## 3. Results and discussion

As mentioned, pardaxin is a pore-forming AMP with two helices (helix I or N-terminal hydrophobic helix and helix II or C-terminal amphipathic helix) and two cationic residues (Lys 8 and Lys 16) [35,38]. In this section, first, the effects of model phospholipid membranes on the structural and dynamical changes of the peptide are investigated. Then, the responses of the lipid bilayers to the peptide and structural changes of the membrane models are studied. Finally, the interactions between the peptide and the lipid bilayers are explored.

### 3.1. Structural and dynamical changes of the peptide

#### 3.1.1. Tilting motions

Previous studies have indicated that transmembrane helices in DMPC bilayers after a few nanoseconds of simulations are tilted up to 30° with respect to the membrane normal direction (Z axis) [60,61]. As well as, previous solid-state NMR studies have shown that bombolitin II (BLT2), a 17 residues long hemolytic peptide with an alpha helical structure, is tilted by 33° to the DPPC bilayer z-direction [62] and the helical axis tilt of the peptide over 20 ns of MD simulation is up to 51° [63]. The solid-state NMR investigations on dynorphin in the DMPC bilayer have shown a tilt angle of 21° [64] and MD simulation studies have also determined a value of approximately 50° [65]. It has been shown that the tilting of melittin in the DPPC bilayer, a peptide with nearly the same structure as pardaxin, gradually increases up to ~25° during 500 ps of MD simulation [66]. Therefore, we investigated the total motions of pardaxin into all six of the model membranes by measuring the average tilt angles of the peptide during last 100 ns of trajectories. The results revealed that during the first steps of MD simulations, the peptide tilted approximately 9° in the DMPC bilayer and approximately13° in the DPPC bilayers, in compliance with tilting of melittin [66]. During the rest of the trajectories, the tilting of the amphipathic helix was constantly increased such that the average of tilt angles over the last 100 ns of MD simulations was 59.64° ± 4.15° and 60.10° ± 3.58° in the DMPC and DPPC bilayers, respectively (Fig 1). These significant differences compared to the aforementioned peptides, can be related to the short simulation times compared to the present study. It seems possible that the larger tilting angles of the peptide appear with the increasing MD simulation times. The tilting angles of pardaxin in the gram-positive and gram-negative bacterial model membranes were increased with increasing simulation time. The results indicated that the average of tilt angles during the last 100 ns of the trajectories was approximately 64.62° ± 8.70° and 59.11° ± 6.11° for the gram-positive and gram-negative model membranes, respectively.

Interestingly, pardaxin nearly maintained its initial orientation in the POPC bilayer and compared to the DMPC, DPPC, and the bacterial model membranes, had a small tilting angle in the POPG bilayer (Fig 1). Among the phosphatidylcholine bilayers, in DMPC and DPPC with the coequal acyl chains, the peptide showed the same behavior, but the overall motions of the peptide in the POPC and POPG bilayers were not similar to each other. Additionally, the tilting angles of pardaxin in the bacterial model membranes were larger than those of in the POPG bilayer. It may be related to the presence of the POPE lipid molecules in the bacterial model membranes. Therefore, there are stronger electrostatic interactions between the peptide and the lipid headgroups in the pure POPG bilayer than POPG/POPE mixed bilayers, as demonstrated in Table 1. These results suggest that the action and orientation behavior of the peptide into the different lipid bilayers depends on both the hydrophobic tail and the headgroup types of lipid molecules, in compatibility with previous experimental studies [35,48,67].

**Table 1:** The electrostatic interactions (kJ/mol) between pardaxin charged residues and the membrane models.

System	Residue			
	Gly1	Glu33	Lys8	Lys16
DMPC	-75.452	-94.082	-55.286	-66.284
DPPC	-96.554	-175.653	-113.211	-102.072
POPC	-101.873	-150.664	-86.875	-103.243
POPG	-724.974	+1392.492	-711.041	-757.123
POPG/POPE (3:1)	-649.067	+1003.981	-655.817	-646.357
POPG/POPE (1:3)	-284.499	+261.978	-275.518	-255.150

#### 3.1.2. Structure and stability of pardaxin

**Fig 2:**
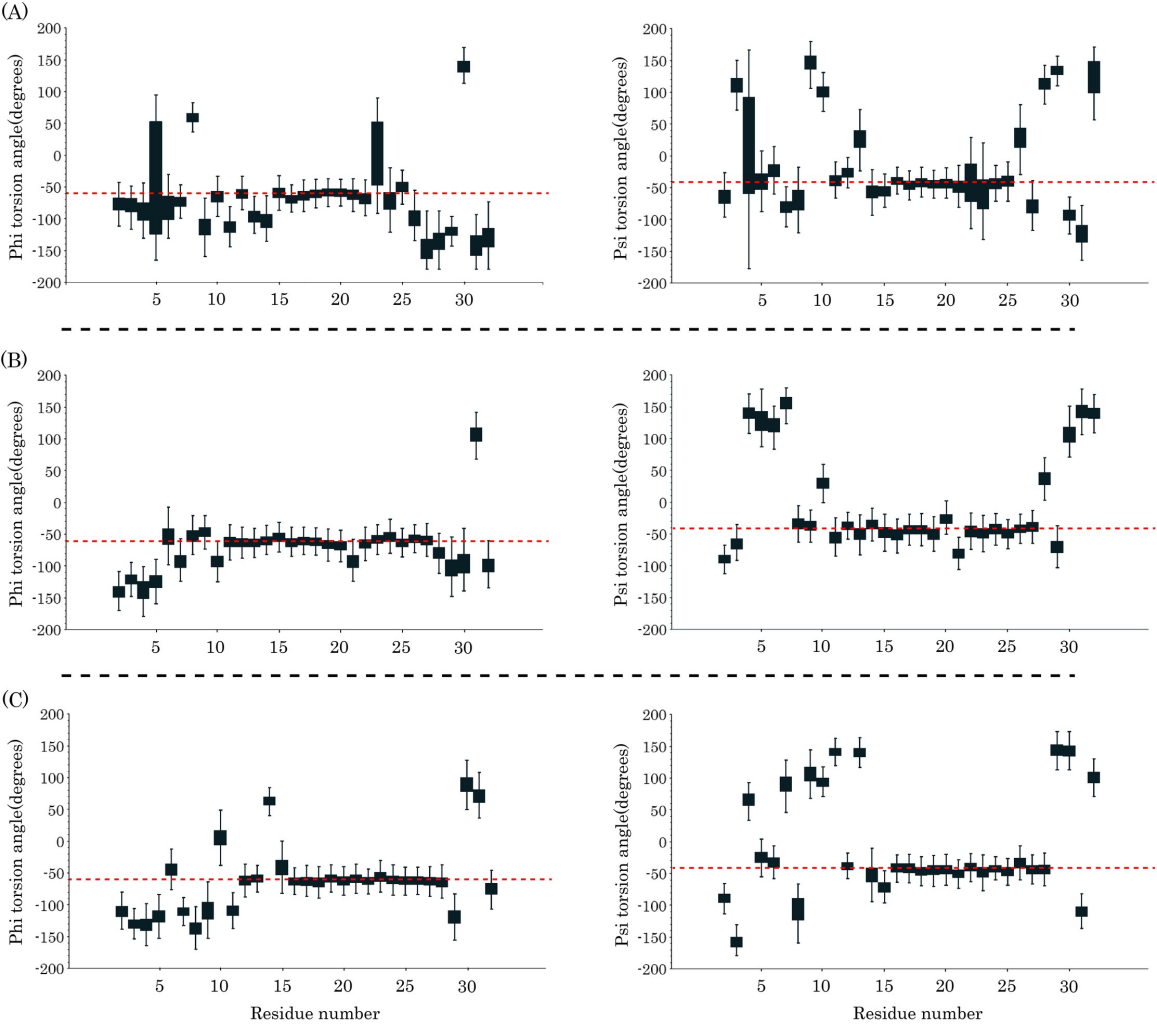
**Represents the torsion angles of pardaxin residues in the (A) DMPC, (B) DPPC, and (C) POPC**. Red dashed lines in the left and right columns show -60 and -45 values, respectively.

**Fig 3:**
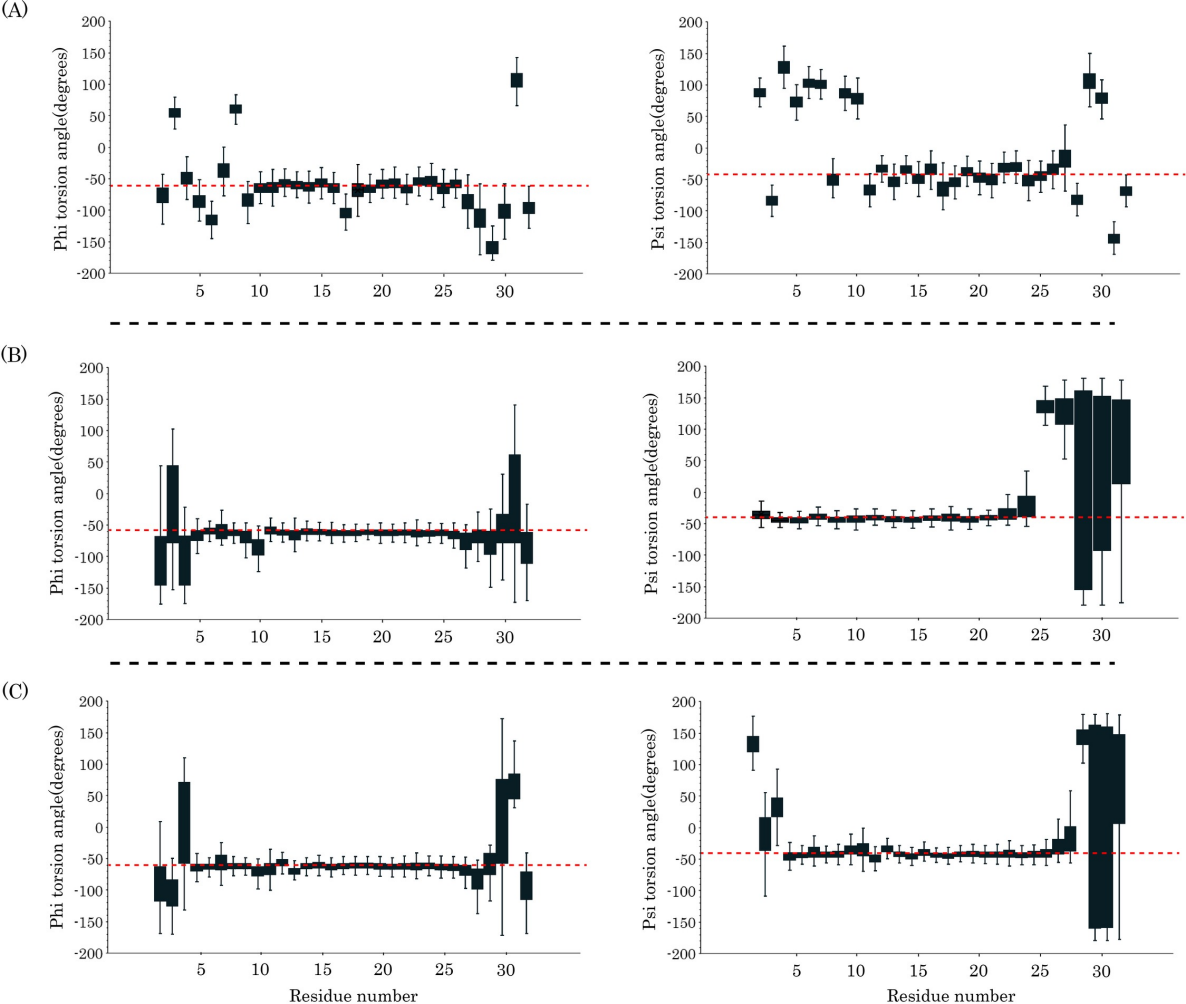
**The torsion angles of pardaxin residues in the (A) POPG, (B) POPG/POPE (3:1), and (C) POPG/POPE (1:3)**. Red dashed lines in the left and right columns show -60 and -45 values, respectively.

Previous circular dichroism (CD) studies have shown that the helicity of pardaxin is increased in the presence of liposomes or synthetic lipids [35,41]. Kolusheva *et al*. indicated an increase in the helicity of pardaxin in the presence of the dimyristoylphosphatidylglycerol (DMPG) and dimyristoylphosphatidylethanolamine (DMPE) bilayers [38]. NMR studies by Porcelli *et al*. have also shown an alpha helical conformation for the C-terminal amphipathic domain of the peptide in both phospholipid bilayers and DPC micelles [35]. Furthermore, it has been shown that when pardaxin bound to the bilayers [34] or lipopolysaccharide (LPS) micelles [39], the peptide rearranged the alpha helical contents and increased its helicity. We characterized the secondary structure distribution of the peptide in the bilayers by backbone torsion angles of the peptide (ϕ and ψ). The analyses demonstrate that generally the N-terminal helix (5–10 residues) in all six types of the membrane was not stable and the majority of values for both ϕ and (/or) ψ angles were further from -60 and -45 values, respectively (Figs 2 and 3). Besides, the same behavior can be observed for the C-terminal residues of the peptide inasmuch as a non-helical conformation is the native structure of this portion. The C-terminal helix (15–25 residues) had the more stable secondary structure in the bilayers and the vast of the ϕ and ψ values were in vicinity of -60 and -45 values, respectively (dashed line in Figs 2 and 3). It is noteworthy that the helical structures of pardaxin were more stable in the POPG/POPE mixed bilayers than the others. The average standard deviation of the phi torsion angles for this region was ±14.17, ±9.92, ±9.89, ±10.90, ±7.09, and ±6.95 in the DMPC, DPPC, POPC, POPG, gram-positive, and gram-negative model bilayers, respectively. This value for the psi torsion angles was ±13.83, ±10.54, ±9.29, ±11.33, ±6.70, and ±6.66 in the DMPC, DPPC, POPC, POPG, gram-positive, and gram-negative model membranes, respectively. Additionally, each box plot in the C-terminal helix region had a short length, indicating that there are the ϕ and ψ values with low dispersion over the 500 times sampling (every 200 ps). It also confirms the stability of the C-terminal helix during the last 100 ns of MD simulations.

Brian *et al*. used circular dichroism (CD) to investigate the conformational changes of pardaxin in 2, 2, 2-trifluoroethanol (TFE), dodecylphosphocholine (DPC), dioleoylphosphatidylcholine/dioleoylphosphatidylglycerol (DOPC/DOPG), and DOPC bilayers. They suggested that the peptide shows a decrease in the alpha helical content in the presence of the lipid molecules [48]. Possibly, this reduction is related to the unstructured N-terminal helix of pardaxin in the model membranes. Pardaxin belongs to the pore-forming neurotoxin groups with an ability to make voltage dependent ion-selective channels in plasma membranes [32,34]. The C-terminal helix of the peptide is crucial to form ion channel and ion exchanging, leading to disrupt the ionic hemostasis and physiology of the target cells. In addition, the N-terminal helix is important to the binding, insertion, and aggregation of the peptide in the target membranes [37,38,41,68,69]. Therefore, stability of the C-terminal helix can corroborate the part of this segment to the ion channel formation by pardaxin. At longer time scales, the N-terminal helix might be rearranged its secondary structure.

#### 3.1.3. Residue snorkeling

**Fig 4:**
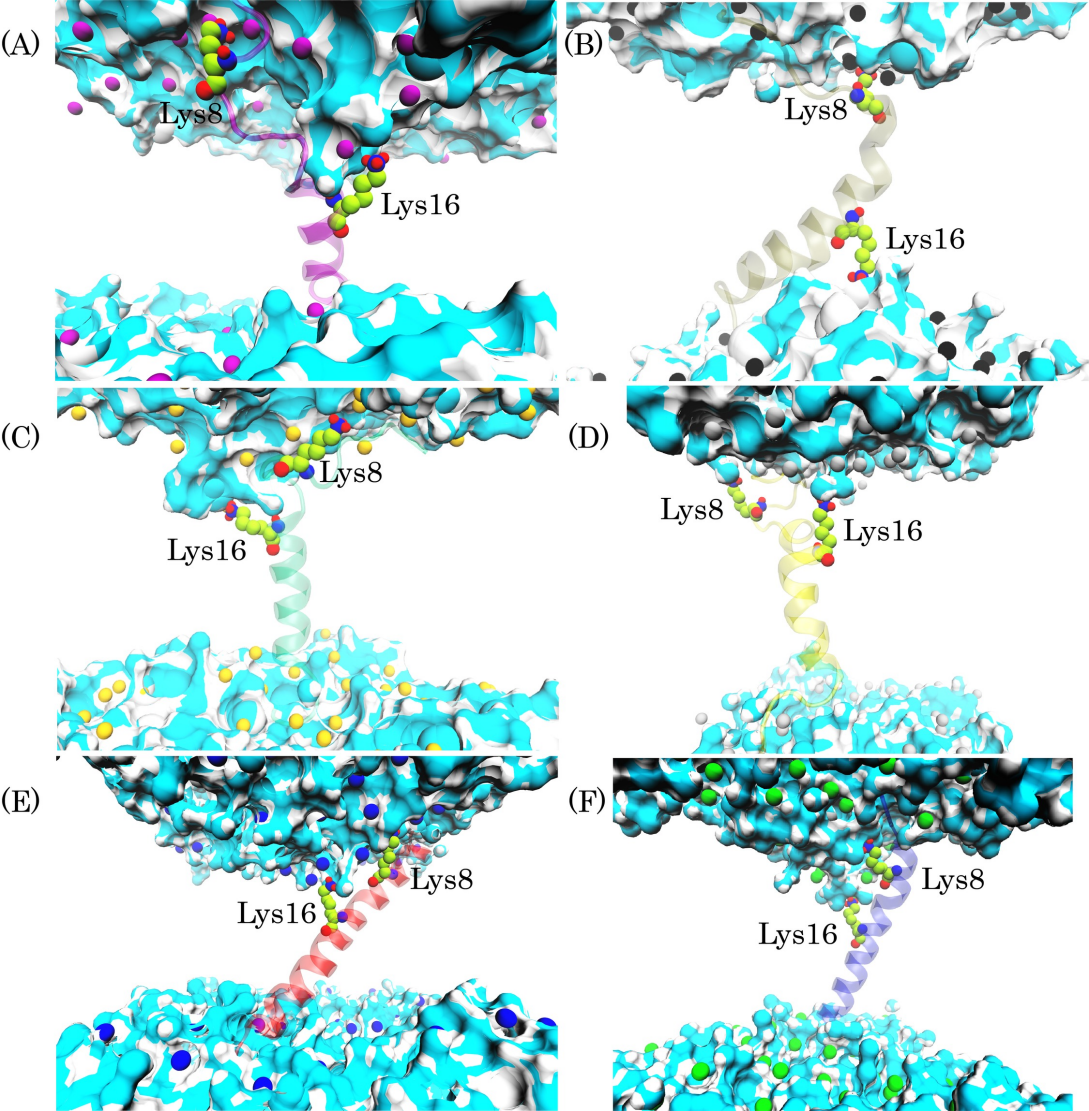
Snorkeling of Lys8 and Lys16 side chains toward membrane-water interface in all six types model membrane. (A), (B), (C), (D), (E), and (F) represent last snapshot of trajectory in the DMPC, DPPC, POPC, POPG, POPG/POPE (3:1), and POPG/POPE (1:3) systems, respectively. Phosphorus atoms and Lys residues are shown as CPK model. The pardaxin structure is shown as a cartoon model. The bilayer acyl chains are omitted for clarity and water molecules are shown as surface model.

Both experimental and simulation studies have shown that the cationic residues defect membranes to retain a hydration shell around the charged side chains [1–9,70]. Fig 4 demonstrates that the Lys8 and Lys16 side chains pulled the phosphorus headgroups of all six types of model membranes and water molecules penetrated into the hydrophobic core of lipid bilayers. The long hydrocarbon side chain of lysine residues provides the ability for the amine group to snorkel toward the membrane interface [6]. As demonstrated in Fig 3, in all lipid bilayers, the amine group of lysine residues was directed toward the bilayer interface and the hydrocarbon side chain of lysine amino acids was remained in the direction of the membrane hydrophobic core. From Fig 3, it can be concluded that when an antimicrobial peptide completely penetrates into the different lipid bilayers and adopts a transmembrane orientation, the basic residues can snorkel toward the membrane interface and maintain the hydration shell around their charged groups, like transmembrane proteins.

### 3.2. Structural changes of the lipid bilayers

#### 3.2.1. Ordering of the lipid molecules

**Fig 5:**
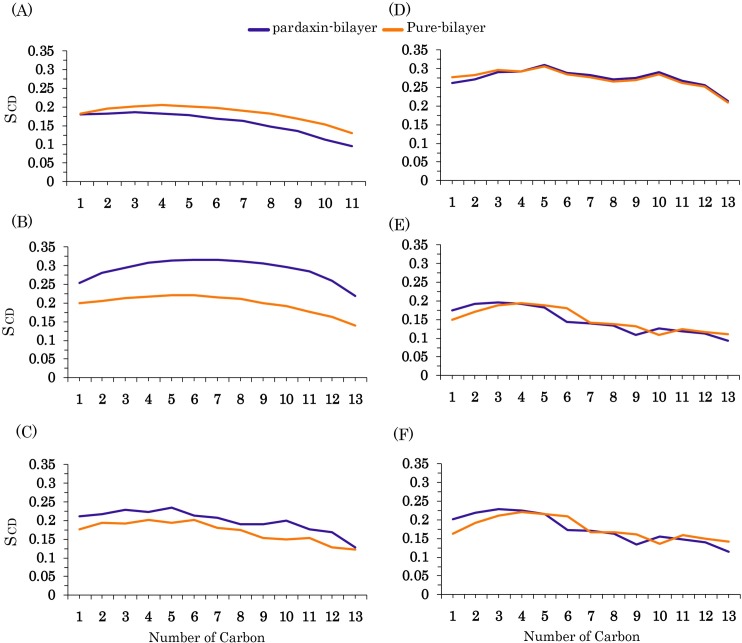
Order analyses. **(A)** Ordering of the hydrophobic tails in the pure lipid bilayer (orange) and pardaxin-membrane complex (violet) for (A) DMPC, (B) DPPC, (C) POPC, (D) POPG, (E) POPG/POPE (3:1), (F) POPG/POPE (1:3) bilayers.

To investigate the effects of pardaxin on the structure of all lipid bilayers, we calculated the deuterium order parameter of the hydrophobic tails in both pure membranes and the pardaxin-membrane complex (Fig 5). As reported in previous experimental studies, the peptide in vesicles composed of phosphatidylcholine (PC) has a transmembrane orientation and disrupts the bilayers using the barrel-stave mechanism [27,48,68]. In contrast, pardaxin has a parallel orientation to the surface of the anionic phosphatidylglycerol (PG) vesicles and the mode of action of the peptide is the carpet mechanism [38,48]. Previous studies have also revealed that the peptide has approximately a surface orientation in the POPC lipid bilayers at low pH (pH = 4.5) and disrupts the membranes with the carpet mechanism [35,67]. In compatibility with these studies, Fig 4 indicates the deuterium order parameters for the DMPC, DPPC, and POPC hydrophobic tails were significantly affected by pardaxin. As demonstrated, the peptide did not markedly change the ordering of the POPG acyl chains. Our previous work has also indicated that when pardaxin bound to the surface of the POPG bilayer, it remarkably affects the ordering of the hydrophobic tails [71]. These results could be one of the answers to the question that why pardaxin disrupts the anionic PG bilayer using the carpet model. As can be seen in Fig 5, the ordering of the lipid acyl chains in the gram-positive and gram-negative bacterial model membranes was affected by the peptide more than the POPG bilayer and the lipid tails nearly showed the same behavior in both membrane models. By comparing the ordering results of the model membranes, it can be concluded that the peptide activity depends on both the headgroup and hydrophobic tail types of the bilayers, in agreement with previous experimental studies [35,48,67].

#### 3.2.2. Mass density profiles and mean square displacement (MSD)

**Fig 6:**
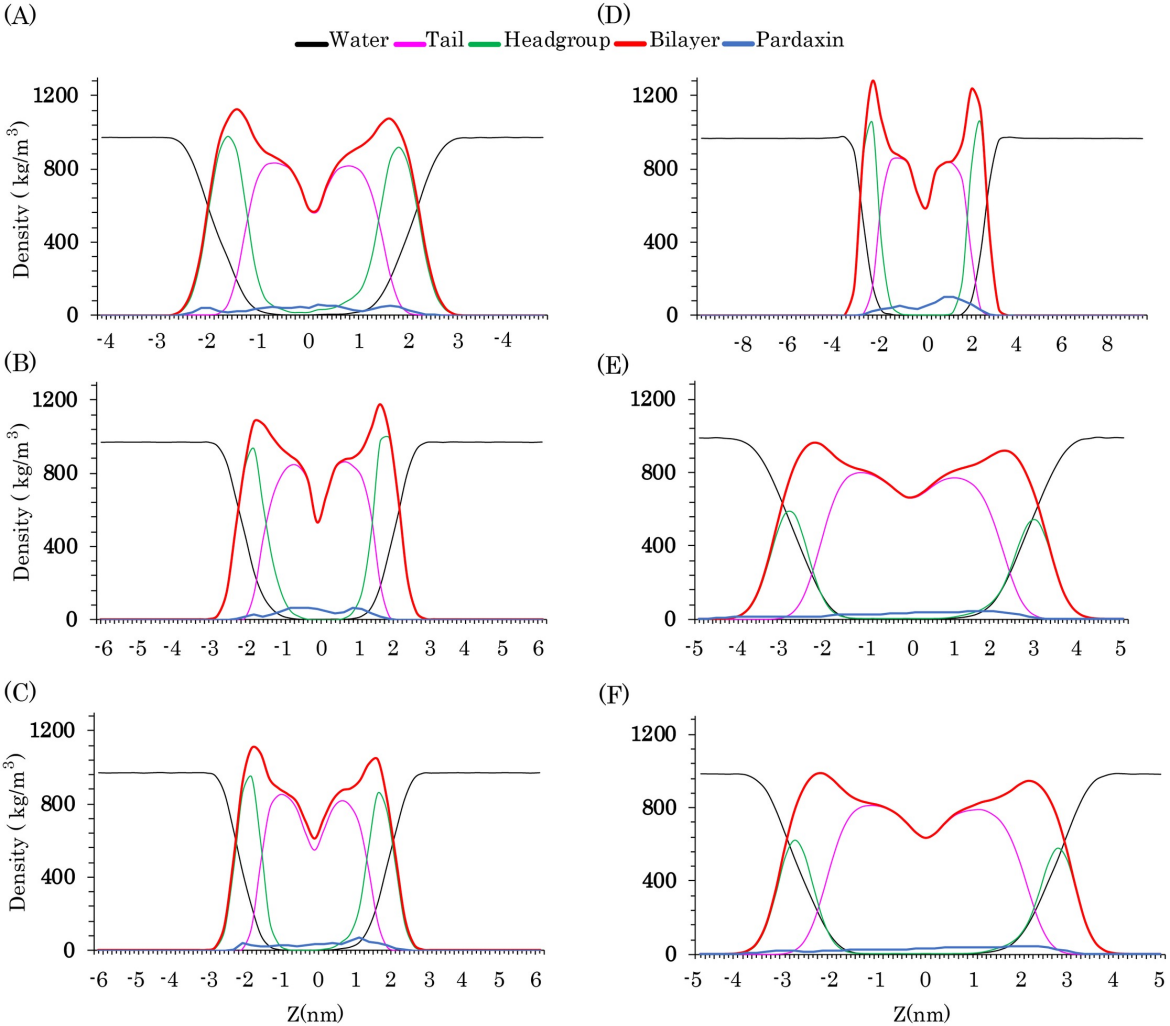
Mass density analyses. Density distribution of the membrane components along the membrane normal direction for (A) DMPC, (B) DPPC, (C) POPC, (D) POPG, (E) POPG/POPE (3:1), (F) POPG/POPE (1:3) simulation systems.

Mass density profile can be used to identify atomic or molecular distributions of lipid bilayer along a specific axis. To evaluate bilayer structure changes, the mass density profile plot of the bilayer components along the membrane normal direction was also calculated. Fig 6 indicates that in all six types of membranes the peptide had a suitable distribution in the top and bottom leaflets. As indicated, in the DMPC, POPC, POPG, and the bacterial membrane models, the density distribution of the upper leaflets was slightly decreased. This is probably because, in high concentrations of pardaxin, when a single form of pardaxin penetrates into lipid bilayers, the N-terminal affects the upper leaflet and facilitates insertion of the other peptides into the membrane of target cells. In contrast, in the DPPC bilayer, the density distribution of the upper leaflet was higher than that of the lower leaflet, suggesting that the C-terminal portion of the peptide affects the structure of model membranes more than the N-terminal portion.

**Fig 7:**
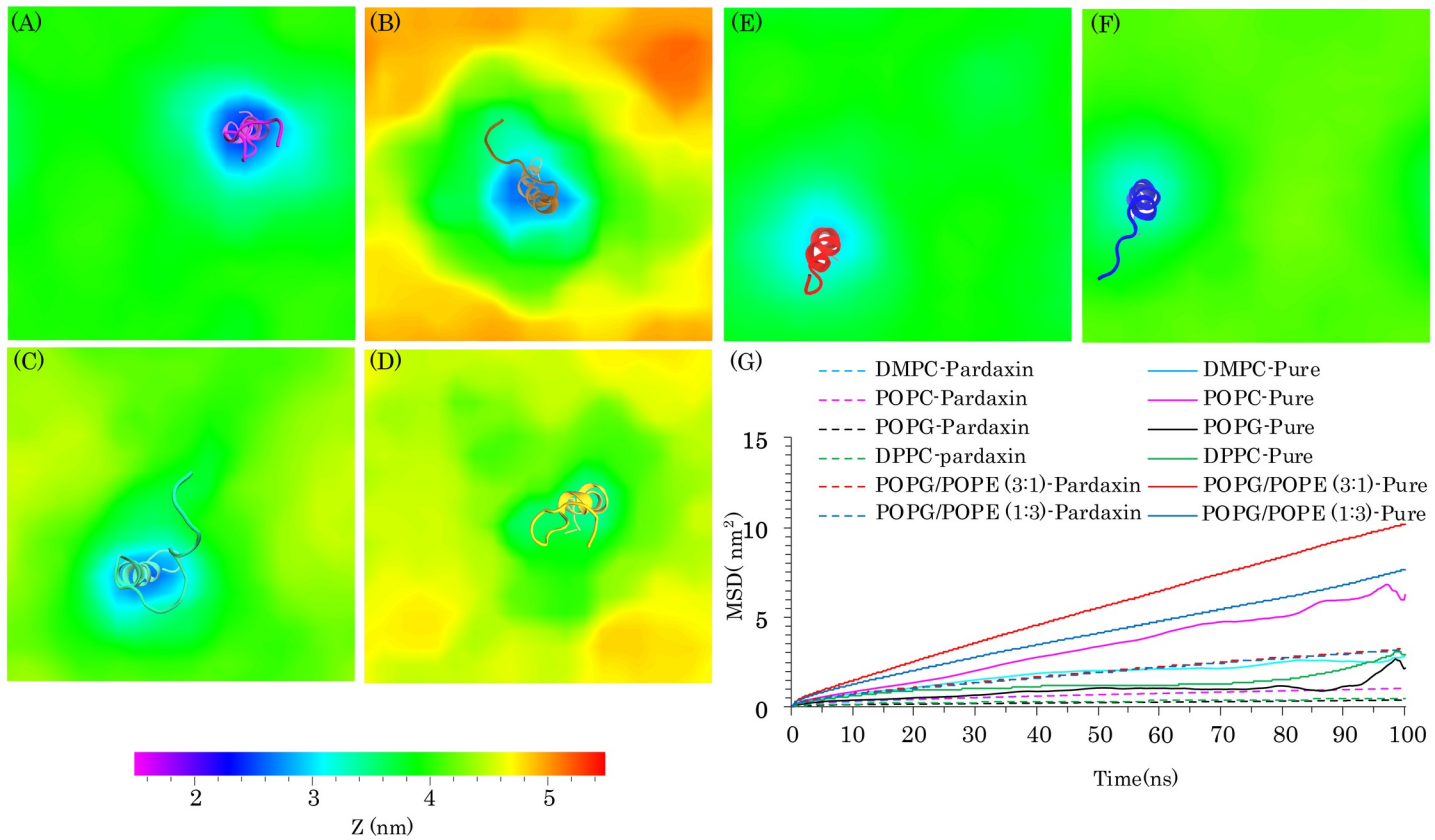
The average thickness of the membrane models and mean square displacement (MSD) of the lipid molecules. **(A), (B), (C), (D), (E), and (F)** Represents the average thickness of all membrane models over the last 150 ns of the MD simulations for DMPC, DPPC, POPC, POPG, POPG/POPE (3:1), and POPG/POPE (1:3) bilayers. The average structure of pardaxin during the last 150 ns of MD simulations is shown as cartoon model for each system. **(G)** Demonstrates MSD of the pure lipid bilayers (filled line) and pardaxin-membrane complex (dashed lines).

Mean square displacement (MSD) analysis provides information on the lateral diffusion coefficient changes of the lipid molecules. Therefore, to measure the effects of pardaxin on the lateral diffusion coefficient of the phospholipids, we calculated MSD for both pure bilayer and the pardaxin-bilayer complex (Fig 7G). As demonstrated`, the lateral diffusion rates of lipid molecules in all pardaxin-membrane complexes were reduced because the movements of phospholipids were restricted by the peptide.

#### 3.2.3. Bilayer thickness

Previous experimental and simulation studies have indicated that the thickness of membranes around embedded peptides is decreased [72–74]. Previous NMR studies have also revealed that bilayers containing zwitterionic phospholipids are remarkably disrupted by pardaxin [67]. To quantify the effects of pardaxin on the thickness and structure of the membranes models, the z-direction distance between the phosphorous atoms was calculated during the last 150 ns of the simulations (Fig 7). The average structure of the peptide was also computed for each system over the last 150 ns of the trajectories. As can be seen from Fig 7, the membrane thickness around the peptide was markedly reduced in the DMPC, DPPC, and POPC bilayers. The most structural deformation was devoted to the DMPC bilayer, which the bilayer thickness in vicinity of the peptide was decreased approximately up to 2nm. Hallock *et al*. revealed that in the presence of anionic phospholipids the ability of pardaxin to disrupt the membrane is decreased [67]. As mentioned, the bacterial membrane models in the present study are composed of the anionic POPG and zwitterionic POPE lipid molecules. Fig 7 (panels A to F) indicates that the deformation of the PC bilayers was more than the POPG/POPE mixed bilayers. This is because of the anionic POPG lipid molecules existing in the bacterial membrane models. Additionally, pardaxin could not significantly deform the pure POPG bilayer (Fig 7D).

As mentioned, experimental studies indicated that the mode of action of the peptide on the PC and PG bilayers are the barrel-stave and carpet mechanisms, respectively. The order and thickness analyses, in good agreement with these results, also revealed that the peptide significantly affected the structure of PC membranes. It can be suggested that when the peptide penetrates into the PC bilayers, it can markedly deform the structure of membrane and facilitate the insertion of the other peptides. In contrast, in the PG bilayers, pardaxin could not remarkably affect the structure of bilayer, suggesting that the peptide prefers to remain on the membrane surface and this is probably the reason of using the carpet model for disruption of the anionic bilayers. The thickness results for the POPG/POPE (3:1) mixed bilayer were nearly similar to the POPG/POPE (1:3) mixed bilayer and the peptide disrupted both mixed membranes more than the pure POPG bilayer. It seems that the presence of POPE phospholipids can increase the membrane perturbation by the peptide. These results are a confirmation of the fact that the peptide can disrupt the zwitterionic membranes more than anionic membranes.

### 3.3. van der Waals and electrostatic interactions between pardaxin and model membranes

As indicated in previous studies, the N-terminal portion of pardaxin strongly binds to the lipid bilayers and the residues in range of 27–33 are important for the pore activity and pore-binding of the peptide [34,35,67,71]. Previous studies have also shown that Pro13 is a crucial residue for the toxin activity of pardaxin [38] and an active pore of the peptide forms by 8 [34] or approximately 6 [75] monomers of pardaxin. Such that the hydrophobic face of the C-terminal helix is associated with the membrane hydrophobic core and the hydrophilic face makes the channel lining to pass ions through the membrane [32,34].

**Fig 8:**
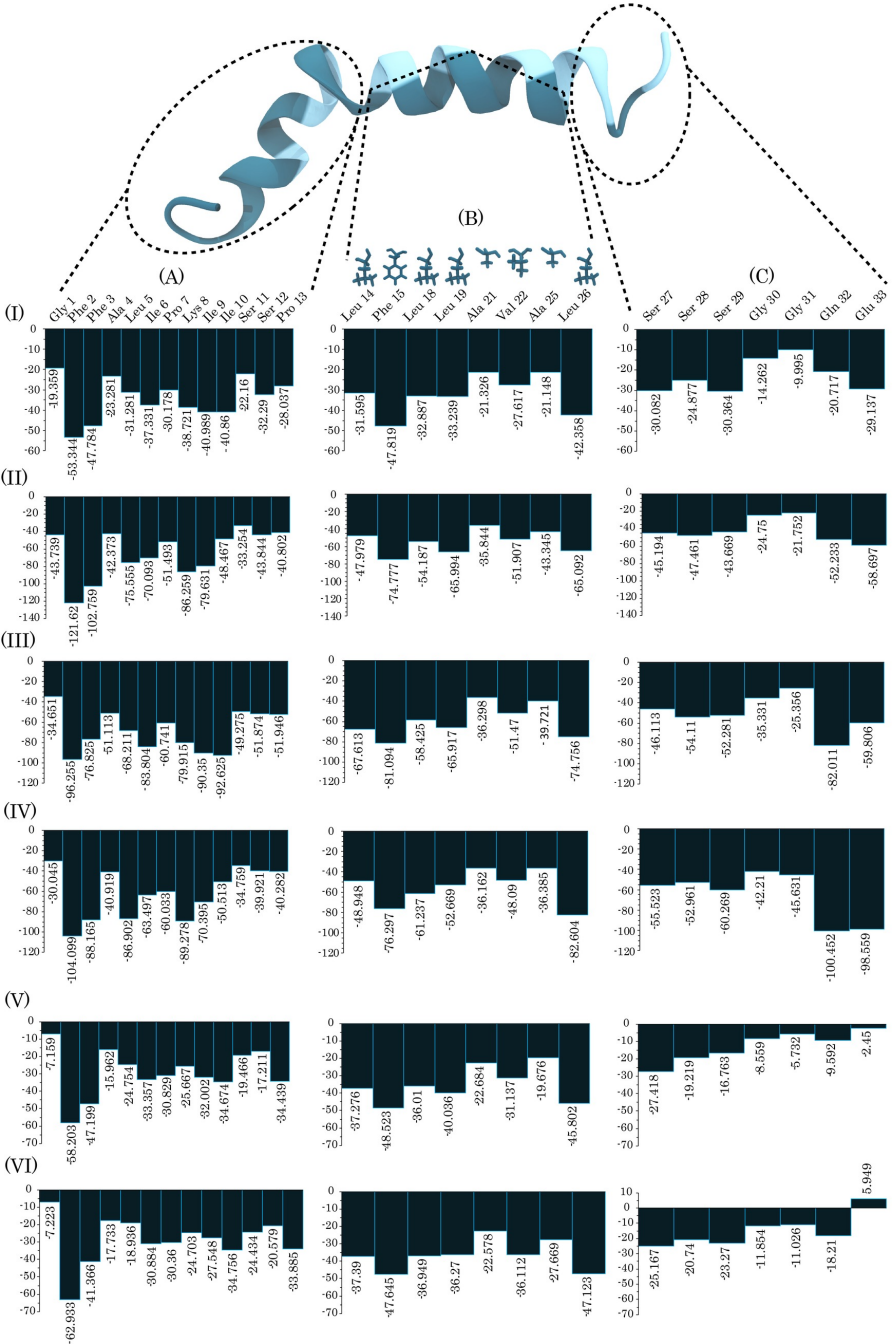
Represents van der Waals interactions between all residues of pardaxin and the membrane models. Pardaxin is shown as a cartoon model (upper) and (I), (II), (III), (IV), (V), and (VI) indicate the van der Waals interactions between each pardaxin residue and the lipid bilayers (bar plots) for DMPC, DPPC, POPC, POPG, POPG/POPE (3:1), and POPG/POPE (1:3) systems. (A), (B), and (C) indicate the amino terminal, amphipathic helix, and carboxy terminal regions of pardaxin, respectively. Hydrophobic and hydrophilic residues of pardaxin are colored as teal and cyan in the cartoon model. Each hydrophobic residue side chain of the amphipathic helix is shown in (B) section. The label numbers represent the van der Waals interaction values.

In general, the family of pardaxins has three structural region; A (1–12 residues), B (14–26 residues), and C (27–33 residues) regions (Fig 8), which are named as amino terminal, amphipathic helix, and carboxy-terminal regions, respectively [34]. We calculated the contribution of each residue of the regions to the total van der Waals (vdW) energies of the peptide using the MM/PBSA method (Fig 8). In the region B, we focused on the contribution energies of the hydrophobic face because it is associated with the membrane core in the cylindrical shape channels of the peptide (Fig 8). This region for P4 has Lys16, Thr17, Ser20, and Ser24 on the hydrophilic face and Ile14, Phe15, Leu18, Leu19, Ala21, Val22, Ala25, Lue26 on the hydrophobic face [34]. As indicated in Fig 8, nearly all residues, including Pro13, were favorable to the vdW interactions with the membrane hydrophobic core.

Previous experimental studies on the engineered peptide 17BIPHE2 have shown that the antimicrobial activity of the peptide against different pathogens is markedly increased by the phenylalanine residues [76,77]. An experimental study has also been reported that the phenylalanine and cationic residues of pardaxin are located at the LPS micelles [39]. In addition, previous simulation studies on the distribution of different amino acids in lipid bilayers have revealed an expansive distribution for the phenylalanine residue in the lipid bilayer regions with a favorable free energy.[78] Brent *et al*. studies on the passive permeation of three aromatic dipeptides through membranes indicated a fastest membrane translocation for the dipeptide containing phenylalanine [79]. As demonstrated in Fig 8, among the residues of the amino terminal region, the phenylalanine amino acids have the effective contributions to the total vdW interactions and Phe2 has the largest contributions in all model membranes. As expected, the contributions of residues to the total vdW interactions were increased with increasing the residue side-chain length. Lys8 had also the significant contributions to the total vdW interactions because it had a side chain with long hydrophobic portion. In the amphipathic helix region, the leucine residues and Phe15 had more interactions with the acyl chains than the other residues. Similar to Phe2 and Phe3, Phe15 had also the effective contributions to the total vdW interactions. In the carboxy terminal region of each system, the contributions of the serine residues were approximately similar to each other. Additionally, Gly30 and Gly31 had the same behavior as the serine residues. In this region for the DMPC, DPPC, POPC, and POPG membrane models, Gln32 and Glu33 had the most contributions to the total vdW interactions than the other residues. These efficient contributions can be related to the longer hydrophobic side-chain (with two methylene groups) than the Ser and Gly residues. These residues in the bacterial membrane models had the vdW energy contribution less than the other bilayers because they were probably further from the hydrophobic core of the membranes. Although, in the DMPC bilayer Gln32 and Glu33 had the effective contributions, their contributions were approximately the same as the serine residues. In general, the results of the vdW energies all suggest that the phenylalanine residues of pardaxin play a crucial role in the peptide-bilayer interactions.

We also calculated the electrostatic interactions between the charged residues of pardaxin and the lipid bilayers (Table 1). As can be seen in Table 1, there were the electrostatic attractions between the zwitterionic headgroups of the PC bilayers and the negatively charged glutamate residue because of the positively charged group, choline, existing in the PC membranes. The positive energy values in the POPG, POPG/POPE (3:1), and POPG/POPE (1:3) bilayers indicated that there was an electrostatic repulsion between the negatively charged glutamate residue and the negatively charged PG headgroups. It is clear that the increased POPG molecule counts in the model membranes also increase the repulsion between the residue and the PG headgroups. The results also demonstrated that there were electrostatic attractions between the N-terminal glycine, because of the amine group of its side chain, and the cationic lysine residues of pardaxin.

## 4. Conclusions

The MD simulation results revealed that in different lipid bilayers, the C-terminal helix of pardaxin was maintained, whereas the N-terminal helix lost its helicity over the 500ns of MD simulation in the most model membranes. Additionally, the ordering of the DMPC, DPPC, and POPC acyl chains are markedly changed by pardaxin. The thickness analyses demonstrated that when pardaxin embedded into the POPG bilayers, the peptide could not significantly affect the membrane structure and stability. The results also revealed that the peptide could affect both the gram-positive and gram-negative membrane models, because of the zwitterionic POPE molecules existing in the membrane models. Furthermore, the effects of pardaxin on the lipid bilayers depend on both the hydrophobic tail and the headgroup types of the target membranes. The lysine residues of pardaxin snorkeled toward the surface of all models of the membrane. In summary, it can be concluded that the phenylalanine residues of pardaxin, either outside or inside of the membranes, play a crucial role on the peptide activity. We hope that these findings will be useful in solving the difficulties in the interactions of membrane-active peptides with the lipid bilayers. Additionally, our findings could contribute to investigations of the interactions between other antimicrobial peptides and the lipid bilayers.
